# Symmetric Connectivity of Underwater Acoustic Sensor Networks Based on Multi-Modal Directional Transducer

**DOI:** 10.3390/s21196548

**Published:** 2021-09-30

**Authors:** Gang Qiao, Qipei Liu, Songzuo Liu, Bilal Muhammad, Menghua Wen

**Affiliations:** 1 Acoustic Science and Technology Laboratory, Harbin Engineering University, Harbin 150001, China; qiaogang@hrbeu.edu.cn (G.Q.); liusongzuo@hotmail.com (S.L.); mohammad.bilalfarooq@gmail.com (B.M.); wenmenghua@hrbeu.edu.cn (M.W.); 2 Key Laboratory of Marine Information Acquisition and Security (Harbin Engineering University), Ministry of Industry and Information Technology, Harbin 150001, China; 3 College of Underwater Acoustic Engineering, Harbin Engineering University, Harbin 150001, China; 4 State Key Laboratory of Ocean Acoustics, Hangzhou Applied Acoustic Research Institute, Hangzhou 311400, China

**Keywords:** UASNs, topology control, directional, connectivity, NP-complete

## Abstract

Topology control is one of the most essential technologies in wireless sensor networks (WSNs); it constructs networks with certain characteristics through the usage of some approaches, such as power control and channel assignment, thereby reducing the inter-nodes interference and the energy consumption of the network. It is closely related to the efficiency of upper layer protocols, especially MAC and routing protocols, which are the same as underwater acoustic sensor networks (UASNs). Directional antenna technology (directional transducer in UASNs) has great advantages in minimizing interference and conserving energy by restraining the beamforming range. It enables nodes to communicate with only intended neighbors; nevertheless, additional problems emerge, such as how to guarantee the connectivity of the network. This paper focuses on the connectivity problem of UASNs equipped with tri-modal directional transducers, where the orientation of a transducer is stabilized after the network is set up. To efficiently minimize the total network energy consumption under constraint of connectivity, the problem is formulated to a minimum network cost transducer orientation (MNCTO) problem and is provided a reduction from the Hamiltonian path problem in hexagonal grid graphs (HPHGG), which is proved to be NP-complete. Furthermore, a heuristic greedy algorithm is proposed for MNCTO. The simulation evaluation results in a contrast with its omni-mode peer, showing that the proposed algorithm greatly reduces the network energy consumption by up to nearly half on the premise of satisfying connectivity.

## 1. Introduction

Underwater acoustic sensor networks are extensively used in marine exploration, disaster warning, sea area surveillance, etc. With the progress of sensor technology and the arousal of marine rights and interests of numerous countries, UASN technology has attracted more and more attention from researchers [1,2,3,4]. However, due to the disadvantages of high propagation delay, low bandwidth and low communication rate brought by the high complexity of underwater acoustic channel [5], transmitting/receiving with a traditional half duplex omni-directional transducer will cause serious packet collision. In fact, many studies [6,7] indicate that a large portion of packet losses are rooted in data conflict, including transmitting/receiving conflict and receiving/receiving conflict [8], which wastes a lot of the very limited energy and reduces the network lifetime.

Some recent works [9] show that using directional technology can greatly improve network performance; this is because the directional transducer [10] is able to radiate toward a certain direction so as to reach farther at the same power, compared with transducer radiating energy omni-directionally. What is more, a long time pursuit for network spatial reuse is achieved by reducing interference between non-communicating nodes in the network, which benefits from the focusing ability of directional transducer. Eventually, it improves the network lifetime as well as the network traffic.

In spite of the huge advantages brought by directional technology, new challenges arise. The first and foremost one is the problem of determining the beam direction to make the total network symmetrically connected [11]. A large number of researchers put forward many different optimization goals with the network connectivity as a constraint, such as the minimum latency data aggregation problem [12], network coverage maximization problem [13], power assignment problem [14], etc. In addition, some authors also make many extraordinary creative works to prove their NP property.

This paper focuses on the challenges that UASNs face with, for example, sound signals attenuate seriously in an underwater environment [15,16], and nodes of UASNs always have limited energy and are almost impossible to be recharged underwater [17]. Studying the problem of maximizing the network lifetime under the connectivity constraint, we intend to orientate the nodes to be connected with minimum energy consumption, which is formulated to a minimum network cost transducer orientation (MNCTO) problem. The problem is reduced from a Hamiltonian path in a hexagonal grid graph problem (HPHGG) [18] that was proved to be NP-complete.

The main contributions of our work are as follows:

1. Based on the tri-modal transducer model, the optimal beamwidth of UASNs stochastically distributed near π/3 is analyzed.
2. For UASNs equipped with π/3 beamwidth directional transducer, the problem of MNCTO is formulated, which is the first attempt, to the best of our knowledge.
3. The problem of MNCTO is proved to be NP-complete by a reduction from HPHGG.
4. A volume model is introduced to denote the energy radiation, especially for omni-directional and directional models.
5. An $O(n^{2})$ complexity heuristic greedy algorithm is elaborated to solve the MNCTO problem.

The rest of the paper is organized as follows: in Section 2, some inspiring related works are introduced; in Section 3, the system model is expounded, including the theoretical analysis of the optimal beamwidth of UADSNs as well as the network model that nodes use to communicate. Then, the problem of MNCTO is formulated, and the NP-complete proof is provided in Section 4; an excellent heuristic algorithm to solve the problem is also elaborated. Next, in Section 5, the simulation evaluations and comparisons are conducted. Finally, the paper is concluded in Section 6.

## 2. Related Works

Connectivity is a very important issue in underwater acoustic directional sensor networks (UADSNs); some related enlightening works are introduced in the following section.

In [19], Li provided a detailed analysis of a cone-based distributed topology-control algorithm, which only depends on the directional information that a node can achieve. The basic idea is to transmit with the minimum power required to ensure that in every cone of degree α around a node *u*, there are some nodes that *u* can reach. It is shown that taking α=5π/6 is a necessary and sufficient condition to guarantee that the network is connected.

In [20], Aschner R. et al. researched, under the condition of an unbounded transmission range, the smallest angle α for which there exists an integer n=n(α) such that for any node set P of *n* antennas of angle α, there can form a symmetric communication graph by appropriately orienting the antennas. Finally, they came to the conclusion of n=4 for α=π/2, and a thesis was further derived, which reveals that if an omni-directional unit disk connected graph meets the condition of $r=14\sqrt{2}$, the induced symmetric communication graph connects.

A new graph structure called α-MST was studied in [21], which is a minimum spanning tree of a set of vertexes P with the additional property that for each point p∈P, the smallest angle around *p* containing all the edges adjacent to *p* is at most α. A generation algorithm is provided in the paper for α-MST, where a boundary of α=π/3 is asserted, while an α-MST does not always exists when α<π/3. In addition, the author reduced the connectivity with α=2π/3 to be NP-hard from HPHGG, whose thinking afforded us a lesson in the proof of MNCTO in this paper.

Ref. [22] studied an interesting relevant geometric problem from the view of mathematics; the authors formulated a problem of building a connected communication network for a set P of *n* vertexes with an α-degree directional antenna, and proved that it is always possible if α≥60. An O(nlogk) complexity algorithm was also proposed to orient the antenna direction, which utilized the properties of the convex hull of P. Nevertheless, given an unbounded transmission range, the network sets up without considering the energy consumption and leads to a geometric topology with a large average link length.

Dobrev S. et al. in [23] conducted some research on the work mentioned above, and showed that for antenna beamwidth α<π/3, the problem is NP-complete to connect the network with a given radius, while for π/3≤α<π/2, it is still unknown how to connect any set of vertexes with a constant range.

Some other related works helpful to our work are summarized as follows Table 1.

Motivated by the observations, including, but not limited to, the results tabulated above, whose ranges seldom cover the condition of α=π/3, which coincides with our previous study on the most suitable beamwidth for an underwater directional transducer, we attempt to conduct some further work on the coincidence brought by the specialty of an underwater acoustic environment, which is illustrated in the next section.

## 3. System Model

In this section, the directional model of UASNs is firstly introduced through the theoretical analysis of the best parameters for the use of a multi-modal underwater transducer, which is called an underwater acoustic directional sensor network (UADSN). Based on the result, the network model and the energy cost model are defined.

### 3.1. Directional Model in UASNs

#### 3.1.1. Multi-Modal Transducer Model

The sound field radiated by multi-modal transducer at different spatial azimuth angles can be expressed as the following formula [10]:

(1) $$p\left(\theta \right)=\frac{B_{0}}{2}+\sum_{n=1}^{\infty }B_{n}\cos\left(n\theta \right)$$

In Equation (1), *p* denotes the sound pressure with reference μPa, θ denotes the azimuth in rad, and $B_{n}$ denotes the weight of the *n*th modal. While cos(nθ), the normalized directional function can be calculated by Equation (2):

(2) $$B_{n}=\frac{2}{\pi }\int_{0}^{\pi }p(\theta )\cos(n\theta )d\theta$$

Therefore, the appropriate parameters can be selected according to the requirements to obtain the intended directivity performance.

Except for the monopole modal (exactly omni-directional mode), most transmitting transducers can also produce dipole modal directivity, and a few may provide the directivity of the quadrupole modal, and thus Equation (1) can be consequently reduced as follows:

(3) $$\frac{p(\theta )}{p(0)}=R\left(\theta \right)=\frac{1+A_{1}\cos\theta +A_{2}\cos2\theta }{1+A_{1}+A_{2}}$$

**Figure 1:**
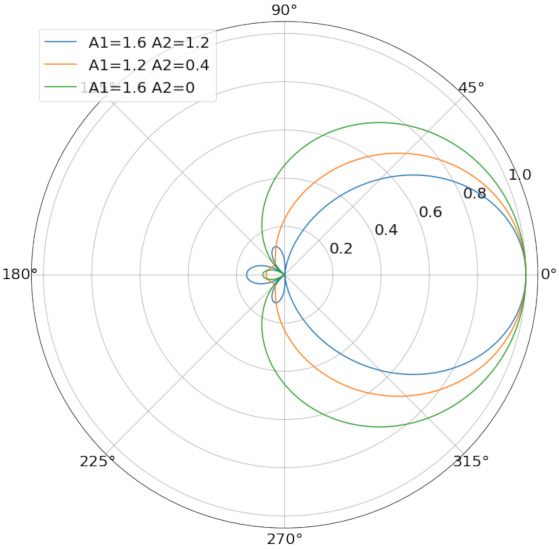
Normalized directional characteristics graphs for different combined coefficients.

For different combination of $A_{1}$ and $A_{2}$, there forms different normalized directional performance, as shown in Figure 1 below:

#### 3.1.2. Optimal Beamwidth

As a rule of thumb, the more communication nodes coveraged by a sponsoring node, the better; the fewer nodes out of its coverage, the better. This ensures directional network connectivity as much as possible. Assuming that some nodes are uniformly distributed in the Euclidean plane and that their coverage area is divided into 8 sectors, the number of nodes nCov(p) that an arbitrary node *p* covers can be denoted by the area that *p* covers, as the formula shows below:

(4) $$nCov\left(p\right)=S\left(-\frac{\pi }{8}<p<\frac{\pi }{8}\right)=\int_{-\frac{\pi }{8}}^{\frac{\pi }{8}}dist(\theta )d\theta$$

(5) $$\begin{array}{cc} nExc(p) & =S\left(\frac{\pi }{8}<p<\pi \right)+S\left(-\pi <p<-\frac{\pi }{8}\right)\\ \; & =\int_{\frac{\pi }{8}}^{\pi }dist(\theta )d\theta +\int_{\pi }^{-\frac{\pi }{8}}dist(\theta )d\theta \end{array}$$

where dist(θ) denotes the farthest distance a beam can reach in direction θ, and Equation (5) denotes the uncovered area of *p*; thus, the node coverage ratio(NCR) can be defined as a ratio of the coverage area of *p* to the uncovered area of *p*, which is denoted by Equation (6) as follows:

(6) $$\begin{array}{cc} NCR(p) & =\frac{nCov(p)}{nExc(p)}\\ \; & =\frac{S(-\frac{\pi }{8}<p<\frac{\pi }{8})}{S\left(\frac{\pi }{8}<p<\pi \right)+S\left(-\pi <p<-\frac{\pi }{8}\right)}\\ \; & =\frac{\int_{-\frac{\pi }{8}}^{\frac{\pi }{8}}dist(\theta )d\theta }{\int_{\frac{\pi }{8}}^{\pi }dist(\theta )d\theta +\int_{\pi }^{-\frac{\pi }{8}}dist(\theta )d\theta } \end{array}$$

Then the goal is to find the parameters that maximize NCR(p). Observing the fact that Equation (6) is closely related to dist(θ), according to sonar equation [27], we achieve the following:

(7) $$\left\{\begin{array}{c} SL(0)-TL(dist(0))-NL=DT\\ SL(\theta )-TL(dist(\theta ))-NL=DT \end{array}\right.$$

In Equation (7), SL(θ) denotes the sound source level in direction θ, TL(dist(θ)) denotes propagation loss, and NL and DT denote the noise level and directional index, individually. Therefore, a further simplification can be made as shown in Equation (8):

(8) SL(0)−TL(dist(0))=SL(θ)−TL(dist(θ))

Consequently, according to the relationship between the sound source level and sound pressure, and the empirical formula of propagation loss in shallow water, we can obtain the following:

(9) $$SL\left(\theta \right)=20lg\frac{p(\theta )}{p_{ref}}$$

(10) TL(dist(θ))=15lgdist(θ)

Substituting Equations (9) and (10) into Equation (8), we obtain the following:

(11) $$dist\left(\theta \right)=dist\left(0\right){\left(\frac{p(\theta )}{p(0)}\right)}^{\frac{4}{3}}=dist\left(0\right){\left(R\left(\theta \right)\right)}^{\frac{4}{3}}$$

So, it is equivalent to solving the following optimization problem, combining the goal as follows:

(12) maxNCR=∫−π8π8(R(θ))43dθ∫−π8π8(R(θ))43dθ(∫π8π(R(θ))43dθ+∫−π−π8(R(θ))43dθ)(∫π8π(R(θ))43dθ+∫−π−π8(R(θ))43dθ)s.t.R(θ)=(1+A1cosθ+A2cos2θ)/(1+A1+A2)0≤A1≤20≤A2≤2

**Figure 2:**
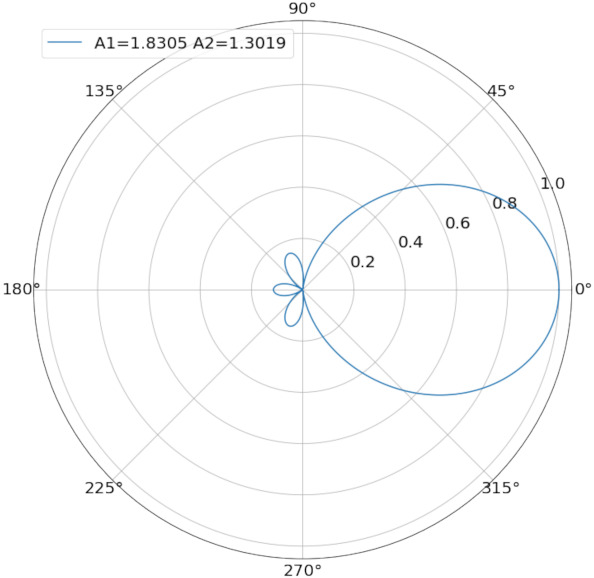
Optimal beamwidth directivity diagram.

Finally, the result of $A_{1}=1.8305,A_{2}=1.3019$ is achieved. The result maximizes NCR(p) and its directional performance is shown in Figure 2.

The corresponding 3dB beamwidth is about $2\theta_{-3dB}=1.23rad=70^{\circ }$, which is just slightly higher than the critical point of α=π/3 that guarantees connectivity, so the fan angle of α=π/3 is chosen as the breakpoint due to the number of beams is an integer and the beams do not overlap each other.

### 3.2. Network Model

In this chapter, the network model is introduced, which models the connectivity properties among nodes. Networks just keep connection among nodes throughout the lifetime, and there are various operating cases, according to their communication modes.

In the omni-directional case, a wireless network is often represented by a connected graph, where vertices correspond to the network nodes, and a directed edge from one vertex *u* to another vertex *v* indicates that data from the node *u* can reach directly to the latter node *v* [28]; the relationship can be expressed as a directed arrow from *u* to *v* as follows:

(13) $$u\to v:\vec{d}\left(u,v\right)<r_{u}$$

The vector $\vec{d}\left(u,v\right)$ denotes the Euclidean distance between nodes *u* and *v*. The directed edge converts to an undirected one if the communication link is symmetrical, i.e., node *v* locates within the transmission range of node *u*, and vice versa, which can be expressed as follows:

(14) $$u↔v:\left\{\begin{array}{c} \vec{d}\left(u,v\right)<r_{u}\\ \vec{d}\left(v,u\right)<r_{v} \end{array}\right.$$

If the nodes share a same transmission range *r*, the equation is simplified to the following:

(15) $$u\overset{r}{↔}v:d\left(u,v\right)<r$$

**Figure 3:**
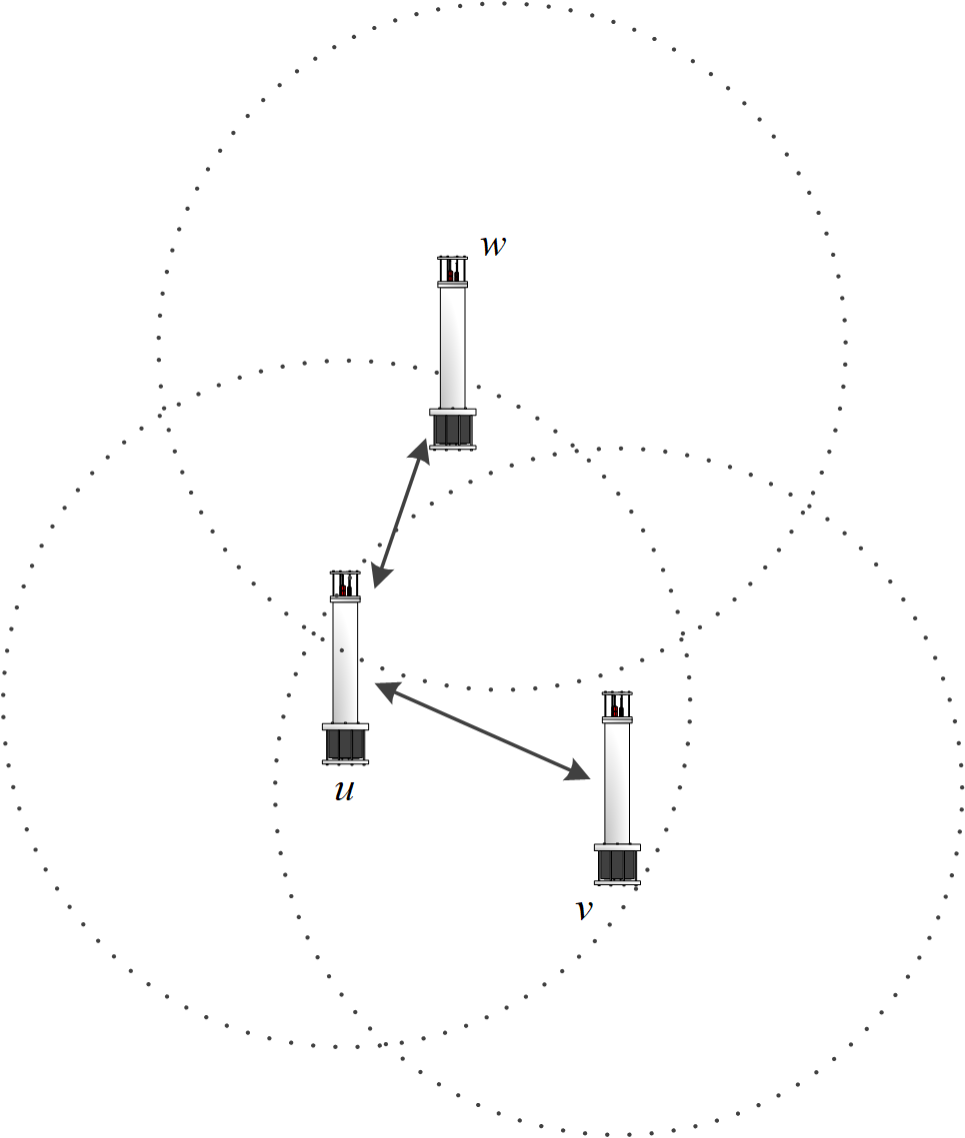
Omni-directional connection between three nodes.

The equation above implies that *u* and *v* are symmetric, connected with transmission range *r*; the relationship of the three nodes sharing a unified transmission range is shown in Figure 3.

In the directional case, it is much more complicated to take into account the situation where two nodes interact because a node can transmit/receive either directionally or omni-directionally. Given a communication mode pair (X,Y), the symbols in the brackets individually represent the transmission and reception modes of a node, so if directivity is considered, there are altogether two modes that the node may work in, as listed below [29]:

- (D,O) ((respectively, (O,D)) mode: A node *u* transmits directionally and receives omni-directionally, or conversely. In this case, *u* can reach any node *v* as long as its beam covers the latter.
- (D,D) mode: A symmetric link sets up if both the sender and receiver are orientated toward each other and they all lay within each other’s transmission range.

**Figure 4:**
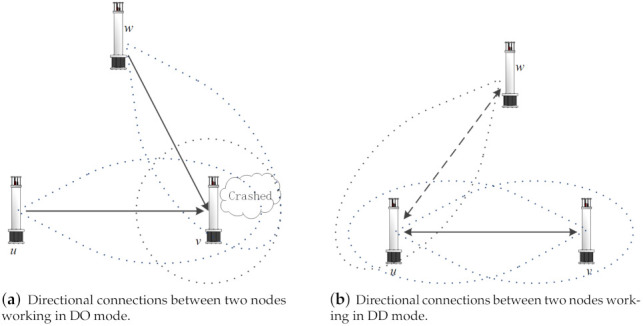
Nodes connection feasibility of different operating modes. Solid lines with arrows represent active communication links.

The diverse link connection feasibility of different operating modes are illustrated in Figure 4, where the solid lines with arrows represent effective communication links, and the dashed ones denote invalid links. As can be seen, Figure 3 is actually a (O,O) mode, where two nodes communicate without any additional limit, apart from the transmission range, while in Figure 4a of the (D,O) mode, a sending node *u* must orientate toward its intended node *v*. If two sending nodes transmit to a same destination simultaneously, a collision then occurs. In the (D,D) mode shown in Figure 4b, two communicating nodes can set up a physical link only if they orientate toward each other at the same time, so node *w* does not influence the ongoing communication between *u* and *v*.

Although it is more convenient to analyze the condition (D,O) or (O,D), the actual network performance may not be as good as that of (D,D), as it is not realistic to reduce the interference between nodes and improve the spatial reusage as desired (see Figure 4b). Thus, the (D,D) mode with beamwidth π/3 is limited in the research.

### 3.3. Energy Cost Model

According to the principle that the multi-modal transducer focuses its beam energy and emits toward a certain orientation spatially, the energy conservation theorem is used for modeling the energy cost model for simplicity. Although it can be modeled as a 2D plane if the nodes depths are identical, we have to take into consideration the actual situation that the multi-modal transducer works in a 3D environment. Thus, the following question is raised: compared with the omni-directional one, how does a transducer with π/3 beamwidth perform regarding energy cost?

**Figure 5:**
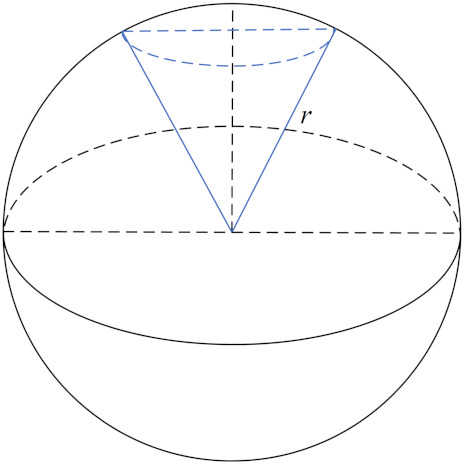
Spatial radiation range of omni-directional and directional models.

Assuming that the radiant energy is uniformly distributed in the space that it covers, the volume of 3D space that a beam covers can be expressed as its energy cost. So the costs for an omni-directional transducer and directional transducer radiating radius *r* are individually denoted as $C_{O}\left(r\right)$ and $C_{D}\left(r\right)$, as depicted below in Figure 5.

We know the volume of the whole sphere is $C_{O}\left(r\right)=\frac{4}{3}\pi r^{3}$, while $C_{D}\left(r\right)$ can be calculated by dividing the volume into two parts, a inverted wedge and a dome. Their volumes can be calculated by a definite integral operation as follows:

(16) $$\begin{array}{cc} V & =V_{wedget}+V_{dome}\\ \; & =\int_{0}^{\frac{\sqrt{3}}{2}r}\pi {\left(\frac{1}{\sqrt{3}}z\right)}^{2}dz+\int_{\frac{\sqrt{3}}{2}r}^{r}\pi \left(r^{2}-z^{2}\right)dz\\ \; & =\frac{\pi }{9}z^{3}{{|}_{0}^{\frac{\sqrt{3}}{2}r}+\pi z\left(r^{2}-\frac{1}{3}z^{2}\right)|}_{\frac{\sqrt{3}}{2}r}^{r}\\ \; & =\frac{\sqrt{3}\pi }{24}r^{3}+\frac{2\pi }{3}r^{3}-\frac{3\sqrt{3}\pi }{8}r^{3}\\ \; & =\frac{2-\sqrt{3}}{3}\pi r^{3} \end{array}$$

Finally, the relative energy cost between the omni-directional and directional models are achieved, and the energy cost ratio (ECR) is defined as follows:

(17) $$ECR_{D}^{O}\left(r\right)=\frac{\frac{4}{3}\pi r^{3}}{\frac{2-\sqrt{3}}{3}\pi r^{3}}=4\left(2+\sqrt{3}\right)$$

This value is approximately 14.93, meaning that directional transducer saves about 93% energy on the condition that they transmit the same distance. The result is used for evaluating our algorithm in the next section.

## 4. Problem Formulation and Algorithm Proposed

In this section, the problem of minimum network cost constrained transducer orientation for the network model discussed above is formulated, and a corresponding algorithm is proposed to solve it.

### 4.1. Problem of Minimum Network Cost Transducer Orientation 

#### 4.1.1. NP-Complete of Transducer Orientation Problem

Firstly, we consider the fixed orientation problem of transducers with π/3, whose orientations are stabilized after the network is established.

Assuming a symmetric connected network composed of nodes set *V* can be expressed as an undirected graph G=(V,E), *E* is the edges set that $\forall u,v\in V,if\;e_{u,v}\in E,u\overset{r}{↔}v$. The nodes are equipped with a directional transducer of beamwidth π/3, and its transmission range is *r*. So the first problem is formulated as follows.

**Problem** **1.** *Given a transmission range R and a set of nodes V with a π/3 beamwidth transducer, can a network be symmetrically connected by assigning to each node a suitable orientation?*

Problem 1 is called the transducer orientation problem (TOP), which is proven to be NP-complete in the following.

**Theorem** **1.** *The TOP is NP-complete.*

**Proof.**  In order to prove that the TOP is NP-complete, we firstly prove TOP∈NP, and then prove that TOP is NP-hard by reducing $HPHGG\leq_{P}TOP$.

For the proof of NP, given a certification $C=\left[C_{1},C_{2},...,C_{n}\right],n=∥V∥$, denoting the set of nodes’ orientations, we can easily determine if the network, including all nodes, is fully connected in polynomial time. Therefore, the TOP is NP.

**Figure 6:**
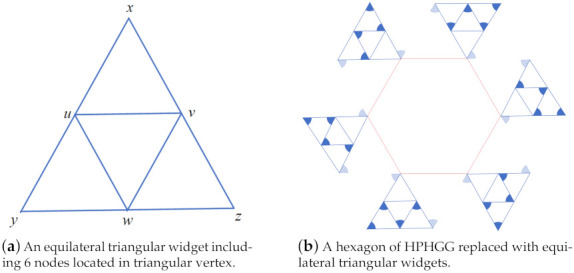
An equilateral triangular widget and a replacement of the hexagon.

Before the reduction is commenced, an equilateral triangular widget of side length *r* is introduced at first. *r* is infinitesimally larger than the transmission range *R*, as is shown in Figure 6a:

The vertexes of the outer triangle x,y,z represent nodes provided with the π/3 beamwidth transducer, and then we add three inner nodes u,v,w located in the midpoint of each edge, so each node can be assigned an orientation so that the triangle vertexes are symmetrically connected, as the triangles in Figure 6b show. We call the vertex in the outer triangle whose beamforms form inward as the EmittingNode, while the other forms as the ContactingNode.

**Figure 7:**
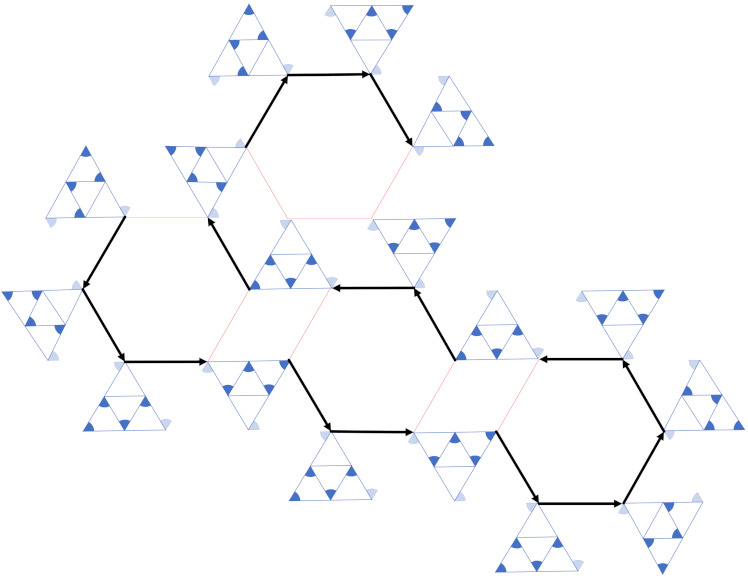
Reduction from HPHGG to TOP.

Each vertex in a hexagon of HPHGG of side length *r* can be replaced with the above widgets in order to construct a reduction, as is depicted in Figure 6b. A connected HPHGG instance with vertexes replaced by equilateral triangular widgets is thus achieved in the same way, as Figure 7 shows, according to Appendix A Lemma A1, in which a few widgets need to be turned round.

From the picture above, we can see that a HPHGG with the largest node degree 3 is found; the paths are highlighted with bold black arrows. In fact, according to [18], a Hamiltonian path can always be found in a hexagon grid graph.

In summary, given a HPHGG, we can reduce it to a TOP by adding some equilateral triangular widgets, which can be completed in polynomial time; thus, the reduction is completed. □

#### 4.1.2. NP-Complete of MNCTO Problem

In underwater environment, the path loss that occurs over a distance *l* for a signal of frequency *f* is given by the following [30]:

(18) $$A\left(l,f\right)=A_{0}l^{k}a{\left(f\right)}^{l}$$

where $A_{0}$ is a unit-normalized attenuation constant, *k* is the spreading factor ranging from 1 to 2, and a(f) is the absorption coefficient. Thus, the acoustic path loss expressed in dB is given by the following:

(19) $$10\log\frac{A(l,f)}{A_{0}}=k\log l+l\log a\left(f\right)$$

Fixing the signal frequency, we can treat the absorption coefficient a(f) as a constant, and the energy cost that a signal travels for a distance of *l* can be denoted in unit dB as the following:

(20) cost(l)=Klogl+Al

where the capitalized letters represent constant coefficients. Then, the problem of MNCTO is formulated as follows:

**Problem** **2.** *Given a set of nodes V with a π/3 beamwidth transducer and a total energy consumption E, can a network be symmetrically connected by assigning to each node a suitable orientation and transmission range R?*

**Theorem** **2.** *The MNCTO problem is also NP-complete.*

**Proof.**  In order to prove that MNCTO is NP-complete, we firstly prove MNCTO∈NP, and then prove that MNCTO is NP-hard by reducing the former proven $TOP\leq_{P}MNCTO$.

For proof of MNCTO∈NP, it can be easily determined whether the network is symmetrically connected under constraint of total cost E in polynomial time. Given a certification of $C=[C_{1},C_{2},...,C_{n}]$, $n=∥V∥$, where $C_{i}=\left[o_{i},cost_{i\to j}\right]$ is a tuple, denotes the cost of node *i* communicating to its adjacent node *j* with orientation $o_{i}$.

In order to reduce $TOP\leq_{P}MNCTO$, we recall the previous structure of HPHGG, whose vertexes are replaced by equilateral triangular widgets in Figure 7 and make a further analysis. Assuming that the side lengths of the triangular and the regular hexagon are identical, *r*, the inner edges of the widgets are of length r/2 and the edges connecting the two widgets are of length *r*, so the according costs are as follows:

(21) $$\begin{array}{c} \begin{array}{cc} cost(r) & =K\log r+Ar\\ cost(\frac{r}{2}) & =K\log\frac{r}{2}+A\frac{r}{2}\\ \; & =K\log r+\frac{Ar-K\log2}{2} \end{array} \end{array}$$

From Lemma A1, we know a widget can only connect to its adjacent peers; in fact, only in this way does it cost the minimum energy. According to the concept of the Hamiltonian path, we obtain the number of edges outside the widgets as n−1, while in order to connect them, the links inside have to be added.

The relationship between number of nodes *n* and the cost of the inner widget is investigated in the following, which is shown in Lemma A2. Thus, the minimum total cost of the whole HPHGG for TOP can be obtained as follows:

(22) $$\begin{array}{cc} \mathrm{COST} & =C_{t}+\left(n-1\right)cost\left(r\right)\\ \; & =\frac{(6n-7)(5K\log r+3Ar-2K)+K\log2}{5} \end{array}$$

Hence, the reduction is conducted. □

Recalling Problem 2, if a TOP has a Hamilton Path, the minimum cost COST is obtained, and if COST≤E, the network can be symmetrically connected by assigning to each node a suitable orientation and transmission range *r*, but not the opposite.

### 4.2. Algorithm Proposed

#### A Greedy Heuristic Algorithm for MNCTO

In order to expound ideas of the algorithm, we analyze and construct a greedy heuristic algorithm to solve the problem of MNCTO step by step in this section.

**Figure 8:**
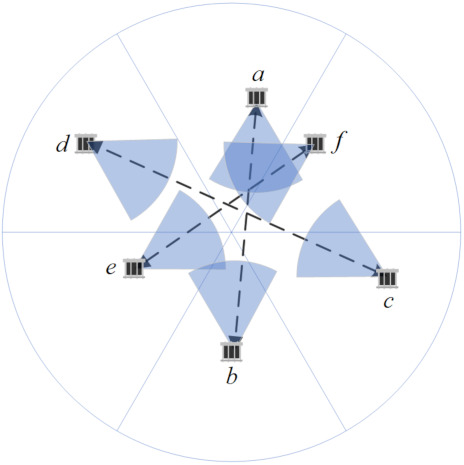
Three pairs of symmetrically connected nodes covering the entire plane.

Given a 2D underwater Euclidean plane randomly deployed of sensors with π/3 beamwidth directional transducer, an intuitive thought is that there must be more than 6 nodes that the whole plane can cover, although the locations and orientations of these nodes should meet some certain conditions. Fortunately, the conditions are not very strict, i.e., even though they need to occupy all the sectors of a disk, the satisfactory orientations are independent of their accurate positions, which just depend on the sector they are located in as Figure 8 shows.

Each node in different sectors can choose its orientation from the directions displayed in Figure 8 to cover the disk center; in this way, its associating node laying in the opposite sector is also connected, and vice versa. The three pairs of nodes are sure to construct three symmetric connections and cover the entire plane.

Obviously, this not enough to build a fully connected network for the reason that we cannot guarantee that the three pair of nodes are interconnected. In fact, this is impossible, according to the lemma bellows:

**Lemma** **1.** *The 6 nodes located in different sectors of a disk cannot interconnect, even if their transmission ranges are unlimited, when they cover the entire plane.*

**Proof.**  It is known from the above analyses that the 6 nodes can form 3 pairs of symmetric connections, and each of them is composed of 2 nodes laying oppositely. Without losing generality, one of the symmetric links a↔b and other link nodes, *e* and *f*, are taken for illustration.

As can be seen from Figure 8, node *b* covers node *f* but not vice versa, because one of their coverage boundaries is parallel. It is the same with node *e*, and thus, a fact can be deduced that nodes in different symmetric connections cannot reach each other simultaneously.

In this way, the lemma is proved. □

Therefore, some more work should be done to construct a core group that guarantees connectivity. Considering the ideas that Paz Carmi proposed in [22], a method to construct the structure is proposed, but first of all, another lemma is introduced as an auxiliary.

**Lemma** **2.** *Any three nodes with directional transducer of π/3 beamwidth can form an interconnection if their orientations are unrestricted.*

**Figure 9:**
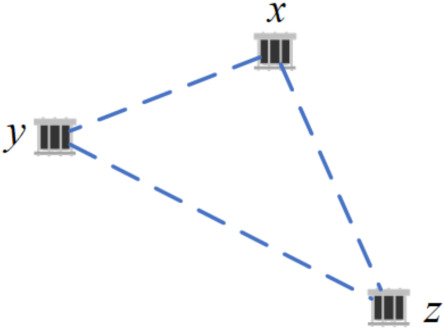
Any 3 nodes with directional transducer of π/3 beamwidth are interconnected.

**Proof.**  Assuming that the three nodes x,y,z compose a triangle whose interior angles are ∠yzx<∠xyz<∠zxy, as Figure 9 shows, despite the special case that it is equilateral (in this case, the three nodes are symmetrically connected, simultaneously), the three angles have the following relationship.

(23) $$\begin{array}{cc} \; & ∠xyz+∠yzx+∠zxy=180{}^{\circ}\\ \Rightarrow \; & 3∠yzx<180{}^{\circ}<3∠zxy\\ \Rightarrow \; & ∠yzx<60{}^{\circ}<∠zxy \end{array}$$

So, there must be an interior angle no more than 60°, assuming it is ∠yzx, which means that the node *z* can cover another two nodes with its π/3 beam, so the symmetric connections between them are easily built only if x,y orientate themselves to *z*. □

Combining Lemmas 2 and Figure 8, it is easy to find a target group composed of at least 9 nodes that always interconnect and radiate omni-directionally, which is called a *target group*. The method is illustrated in Lemma 3 as follows.

**Lemma** **3.** *Given a disk with 6 sectors, if each sector covers at least 1 node with beamwidth π/3, called HexNodes, and another 3 homogeneous nodes, which are called TriNodes that are located in 3 interlaced sectors of them, the nodes interconnect if the maximal interior angle of the TriNodes is no more than 2π/3.*

**Proof.**  For the reason that further constraint of orientations emerge, here, we need to prove that the connections are assured between HexNodes and TriNodes as well as the connections of the inner TriNodes.

First of all, it is known that two HexNodes in opposite sectors are connected, so if we want to connect the 6 HexNodes through TriNodes, there must be one TriNode in each opposite sector. This can be described as a permutation and combination problem, i.e., how we can select from sector sets of [0, 3], [1, 4] and [2, 5] that every set is chosen as the only element? Taking [0, 1, 2] and [0, 1, 5] as examples, both sets construct a semicircle, implying that the three TriNodes lay on one side of the diameter; in this situation, they cannot communicate with all the HexNodes. Thus, there are only two possible scenarios left: [0, 2, 4] and [1, 3, 5].

**Figure 10:**
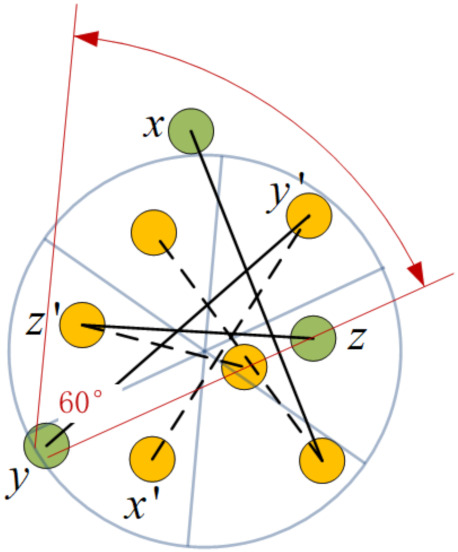
Nine nodes make cross-connections.

As depicted in Figure 10, 9 nodes construct a *target group* satisfying the above conditions, where the dashed lines denote symmetric connections of the inner HexNodes that are colored in orange. The solid lines will be analyzed next. Without loss of generality, let x,y,z be the outer nodes in the sectors, which cover no less than 2 nodes, and the corresponding $x^{\prime },y^{\prime },z^{\prime }$ lay in the opposite sectors. From Lemma 2, we know there must be an acute angle formed by TriNodes:x,y,z, assuming ∠xyz, so node *y* can cover x,z with a π/3 beam as well as node $y^{\prime }$, covered by ∠xyz. On the other hand, *y* lays in the sector covered by $y^{\prime }$; thus, a symmetric connection $y↔y^{\prime }$ and two directed connections, y→x and y→z, are set up.

Meanwhile, assuming that ∠yzx is the maximal interior angle, which is less than 2π/3 and larger than π/3, then $∠xzz^{\prime }+∠z^{\prime }zy<2\pi /3$; this implies that either $∠xzz^{\prime }$ or $∠z^{\prime }zy$ must be less than π/3, so a symmetric connection $z↔z^{\prime }$ and an only directed connection of either z→y or z→x can be established. The same applies for *x*.

Thus, the Lemma 3 is proved. □

Based on the analyses above, an ingenious way to construct a connected group that radiates the entire plane is thus formed, as is detailed in the following algorithm.


**Algorithm 1** MNCTO.**Input:** Nodes set V, Anticipated groups number nGroups**Output:** Target groups set gSet1: **procedure**
Find_Tar_Groups (V,nGroups)2:  nSet←set();                  // Saves isolated nodes set3:  gSet←set();                      // Target group set4:  **if**
nGroups<1
**then**5:   **return**
*⌀*6:  **else**7:   tgSet←KMeans(V,nGroups);       // Clustering V with K-Means8:   **for all**
group∈tgSet
**do**9:    **if**
group contains more than 9 nodes **then**10:     c←CalculateCentroid(group);     // Get the centroid of the group11:     g←group;12:     partition *g* into 6 sectors;13:     **if**
*g* has a target group gItem
**then**14:      gSet←gSet∪gItem;15:      nSet←nSet∪(g−gItem);16:     **else**17:      Sorting gGroup with asending order for all its vertexes18:      **for all**
vertex∈group
**do**19:       angle←CalculateAzimuth(c→vertex);20:       g←Rotate(g,angle);     // Rotate *g* with angle anti-clockwise21:       **if**
*g* has a target group gItem
**then**22:        gSet←gSet∪gItem;23:        nSet←nSet∪(g−gItem);24:        break;25:       **end if**26:      **end for**27:      nSet←nSet∪group;28:     **end if**29:    **else**30:     nSet←nSet∪group;31:    **end if**32:   **end for**33:   **if**
len(gSet)=0
**then**              // In order for convergence34:    nGroups←nGroups−135:   **else**36:    nGroups←nGroups−len(gSet)37:   **end if**38:   **return**
gSet∪Find_Tar_Groups(nSet,nGroups);    // Recursion39:  **end if**40: **end procedure**


In Algorithm 1, an operation of rotating a group is a method that endeavors to make it a *target group*, whose key idea is transforming a rotating problem of continuous angle to a discrete condition, and thus, the computational expense is upper restricted by the group size without affecting its performance too much. The Algorithm 2 is listed as follows.


**Algorithm 2** MNCTO.**Input:** Target group gGroups, Rotating Angle bias**Output:** Rotated target group gGroup1: **procedure**
RotateA (gGroup,bias)2:  **for all**
vertex∈gGroup
**do**3:   azimuth←getAzimuthAngle(gGroup.center,vertex)−bias+0.00014:   Re-calculate sectorId for azimuth5:   gGroup resets sectorId for vertex6:  **end for**7:  **return**
gGroup8: **end procedure**


Nevertheless, although we have our network cut apart into *target groups* and outliers, it is not completely connected until the connections for the two kinds of nodes are built. Recollect the capability that a *target group* beams omni-directionally; it is easy to set up symmetric connection between a *target group* with an isolated node, because no matter how a *target group* rotates (see Algorithm 1), the outlier must lay in one of its sectors. However, this truth is unavailable for connecting two different *target groups* for the reason that their orientations are fixed. Fortunately, we investigate the problem and reach the conclusion as follows:

**Lemma** **4.** *Given two target groups of nodes stochastically distributed with arbitrary deflection angles in a Euclidean 2D plane, which are composed of nodes with a π/3 directional transducer whose transmission range is unbounded, they are symmetrically connected through at least one link.*

**Proof.**  Without loss of generality, two normal cases are analyzed for the proof.

Given that the radius of the two *target groups* is *R* and the distance between their centers is DIST, we know there must be a value of DIST for which one of the groups is entirely covered by a HexNode of another group. The value is denoted by $D_{t}$; it is dismissed because it does not affect the conclusions. Then, we have two cases of the distance relationship between the two groups.

- Case 1: $DIST<D_{t}$
- Case 2: $DIST\geq D_{t}$

**Figure 11:**
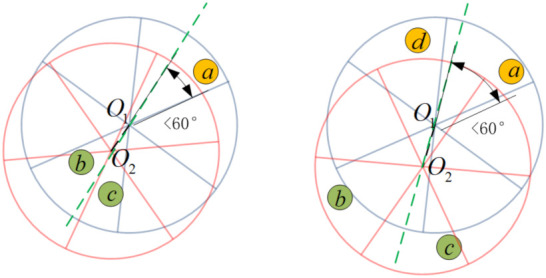
Two target groups are connected in the case of $DIST<D_{t}$.

In Case 1, where the line links the centers $O_{1},O_{2}$ of the two groups and is extended as a guideline L, there must be two HexNodes in one of the groups that are separated, which are assumed to be b,c of the group $O_{2}$ (the disk of red lines) as Figure 11 shows. Obviously, HexNode*b* and *c* may be located in the same sector or different sectors of group $O_{1}$.

As for the former situation, b,c are covered by *a* because L passes through $O_{2}$; together with the reason that the beam boundaries of HexNode are parallel with the sectors boundaries, the coverage area of *b* and *c* is 2π/3. Considering that *a* lies opposite of *b* or *c*, the angle of $O_{2}\to a$ with L must be less than π/3, so *b* or *c* is affirmative to cover *a*.

In another situation in which b,c are covered by *a* and *d* individually, the center $O_{1}$ must be covered by either *b* or *c*, which is assumed to be *c*, as Figure 11 shows. With the help of guideline L, we find that the included angle $aO_{1}$ with L is less than π/3, so *a* is sure to be covered by either *b* or *c*.

In this way, the validation that two *target groups* are connected in the case of $dist<D_{t}$ is proved.

While in Case 2, we know that all HexNodes of a *target group* are covered by a single HexNode, the situation will be the same with the first situation of Case 1, and therefore, they are connected too.

Thus, we prove the truth of Lemma 4. □

For the last step, we just need to set up links between the *target group* and between the *target group* and *target group*, as well as links between *target groups* and outliers, as the Algorithm 3 describes below:


**Algorithm 3** MNCTO.**Input:** Target groups gGroups, Isolated nodes outliers**Output:** Void1: **procedure**
Establish_Full_Connections (gGroups,outliers)2:  MAXDIST=655363:  gGroups←MST(gGroups)      // Establish MST for gGroups centers4:  **for all**
group∈gGroups
**do**5:   **for all**
tar∈gGroups
**do**           // link *target group* with peers6:    **if**
group not tar
**then**7:     link group with tar8:    **end if**9:   **end for**10:   **for all**
node∈group
**do**         // link TriNodes with HexNodes11:    **if**
node∈TriNodes
**then**12:     link node with its peer       // peer: node locating in opposite sector13:    **end if**14:   **end for**15:   **for all**
node∈TriNodes
**do**         // link TriNodes themselves16:    find node corresponding to the smallest angle17:    link node with other 2 TriNodes18:   **end for**19:  **end for**20:  **for all**
node∈outliers
**do**     // link outliers with their nearest *target group*21:   tGroup←null22:   dist←MAXDIST23:   **for all**
group∈gGroups
**do**24:    **if**
distance(node,group)≤dist
**then**25:     dist←distance(node,group)26:     tGroup←group27:    **end if**28:    link node with tGroup29:   **end for**30:  **end for**31: **end procedure**


In this way, connections for all the nodes in network are established. An artifice of recursion is adopted in Algorithm 1, aiming at finding as many target groups as possible, which guarantees that the algorithm can be maximally energy efficient.

## 5. Simulation Evaluations

In order to provide a detailed performance exhibition of the proposed MNCTO algorithm, several metrics such as the total energy consumption and average hop distance are designed to quantify the algorithm performance; a comparison of the omni-directional mode is provided as reference.

### 5.1. Performance Metrics

- Network Energy Cost ($E_{nc}$)
  The energy consumed by a network G=(V,E) originates from packets delivered from the source node to sink node. In ad hoc networks, all nodes are homogeneous and able to act as at least one of sender/relay/sink, so the metric $E_{nc}$ is defined to evaluate the energy efficiency, as shown below:
  (24) $$E_{nc}=\frac{1}{N}\sum_{i=1}^{N}\sum_{u,v\in V}d_{u,v}^{k},\;u\neq v$$
  where N=1000 random packet paths are counted, u,v in denote arbitrary two stochastic nodes and $d_{u,v}^{k}$ implies that the cost is positively correlated to the spreading distance (see Equation (18)), where *k* denotes the spreading factor that ranges from 1 to 2 in an underwater environment; thus a median of 1.5 is used. In addition, $ECR_{D}^{O}$ needs to be weighted when compared with the omni-directional mode, and $d_{u,v}$ should be normalized by a common constant; thus the network deployment boundary is chosen.
- Delivery Distance Distribution
  As the term suggests, this metric counts the distribution of traveling distances of large quantities of packets with random source and sink, which is actually another enhanced metric of network energy consumption that presents key performance intuitively.
- Average Single Hop Distance($D_{hop}$)
  It indicates the average link distance, and can be measured as Equation (25), where $d_{u,v}$ denotes the traveling path length of a random packet, while $h_{u,v}$ denotes the hops number between u,v.
  (25) $$D_{hop}=\frac{\sum d_{u,v}}{h_{u,v}}$$

In order to confirm the performance of the algorithm under stable conditions and meet the premise of Lemmas 1 and 4, MNCTO is designed to be a transmission range free algorithm, meaning that the network implementing MNCTO is always connected without much attention given to the parameter of the transmission range, but it is not the same with an omni-directional network modeled as an unit disk graph (UDG), where a communication link between two peers is set up as long as their distance is less than a certain value. A situation that must be guaranteed is the connectivity of the latter, which is still a challenging topic of research so far [31]; thus, a Monte Carlo analytical method is developed. Various situations with different combinations of nodes number and transmission range are simulated to provide comparisons.

### 5.2. Simulation Performance

**Figure 12:**
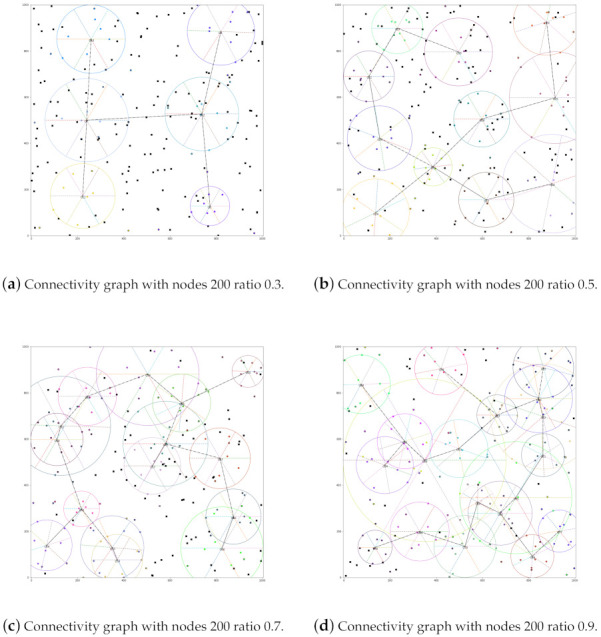
The connectivity graph of the network with 200 nodes.

Given an Euclidean 2D square plane and some nodes randomly deployed within the water area, different metrics between underwater acoustic sensor networks running MNCTO and UDG are evaluated. The simulation parameters are listed in Table 2:

The reason why the clustering ratio (CR) is chosen as a parameter bases on an observation that the recursive MNCTO would try its best to make every 9 nodes *target groups*; nevertheless, it is not always necessary because it may generate groups of large communication radii during the anaphase, as Figure 12 exhibits.

In the figure above, the circle with six sectors denotes a *target group* with a certain rotation angle; this phenomenon is more obvious on occasions of higher ratio value. Solid circles of the same color within a group denote the corresponding HexNodes, while a TriNode is displayed as an isomorphic one with a black edge. The other black squares denote Outliers, which should be connected to their nearest group. At last, the dashed dotted line indicates that the two groups are connected. Not all connections are shown for the simple purpose that the figure is not chaotic.

As can be seen, various numbers of target groups are generated, according to the value of the ratio. Recall that in a network of 200 nodes, the maximum number of groups formed is 22, as, for the greedy MNCTO, it will find as many *target groups* as possible, so the phenomenon will be inevitable in the convergence stage of the algorithm. A simple but effective method is to limit the upper boundary of the intended number of the *target groups*. In fact, with the increase in the number of *target groups*, the average scope that a group covers decreases as a trade-off, so the gap is not as obvious as imagined.

**Figure 13:**
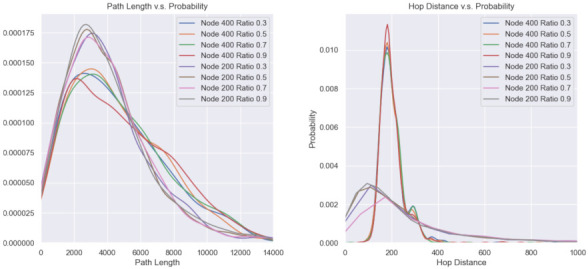
Path length distributions for different node numbers and ratios.

Other performance indicators, such as the delivery distance distribution and average hop distance, are provided below in Figure 13. It can be seen from the left of the figure that the curve with a higher number of nodes is more dumpy, indicating that with an increase in nodes, the total path length tends to be uniform. The right of the figure shows a mutually corroborating conclusion, namely, the too-high or too-low numbers of hops reduce, and the waveform energy is concentrated in a certain value of the distance of 200, meaning that the energy consumption of the nodes is balanced.

**Figure 14:**
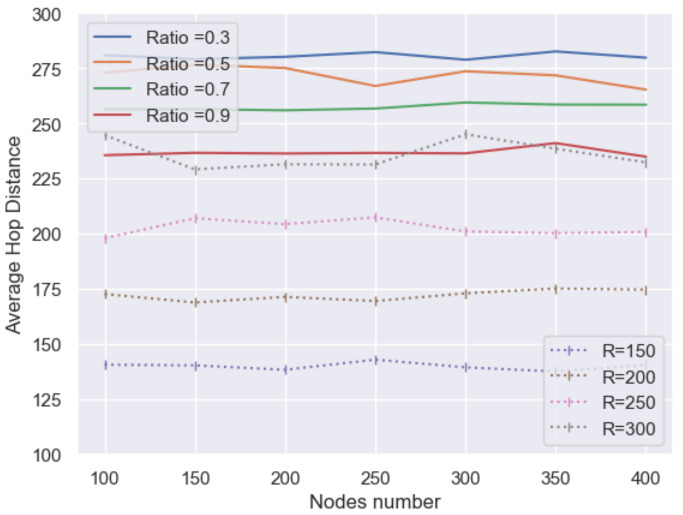
Average hop distance comparison.

**Figure 15:**
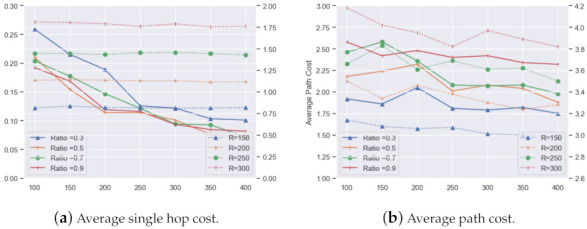
Energy cost of a single hop and a successful delivery.

In Figure 14, the performance of the average hop distance (AHD) between MNCTO and UDG are simulated. It is apparent that with CR increasing, the AHD of MNCTO tends to decrease but is still higher than that of UDG. The reason lies in the algorithm generating a target group that a directional node cannot communicate to its adjacent neighbors directly. While taking the energy cost into account, the former is more energy efficient, as is depicted below in Figure 15a, where the scales are all relative:

What can be seen from the above figure is that the relay process of the directional mode saves as much as 90% energy compared to the omni-directional mode. It also has a better performance of the average delivery cost all over the whole network in spite of its higher path length, as Figure 15b reveals. The figure has two scales: the MNCTO is represented by the ordinate on the left, while UDG is represented by the opposite ordinate. A network implementing MNCTO with appropriate parameters may save more than half of the energy, which is crucial for the underwater environment. Another observation is that, with the expansion of network scale, the average hop cost reduces, the performance differs from Figure 14; the reason lies in the result shown in Figure 13, i.e., the hop distances distributions are more concentrated. Larger CR leads to a longer path distance, neutralizing the advantages brought by short single hop distance; however, it is still comfortably ahead of the omni-directional network.

## 6. Conclusions

From the perspective of the challenges that UASNs are confronted with, such as topology control and energy efficiency, this paper investigates the advantages brought by directional communication technology. However, there are many difficulties involved, among them, the network connectivity being the key challenge to be solved. This research provides a reduction from HPHGG to prove that the issue to connect an UADSN with a π/3 beamwidth directional transducer is NP-complete as well as to further the NP-complete proof of connecting the network with minimum cost, which are defined as the TOP and MNCTO problems, individually. Subsequently, a greedy heuristic algorithm is proposed to construct a connected network of $O(n^{2})$ complexity degree with constant probability 100%; relevant proofs are also provided within the paper. The simulation results show that the proposed algorithm achieves the goal of full connection with lower energy cost, compared with traditional UASNs equipped with omni-directional transducers, which saves more than half of the energy. The research provides some significant guidance for further research in related fields.

## Figures and Tables

**Table 1:** Researches for symmetric connectivity problem under condition of one beam.

Beamwidth	Mode	Range	Complexity	Reference
α<π/3	Fixed Orientation	Not always guaranteed	NPC	[22]
α=π/2	Fixed Orientation	$14\sqrt{2}$	O(nlogn)	[20]
α=π/2	Fixed Orientation	7	O(nlogn)	[24]
π/3<α<2π/3	Fixed Orientation	4/cos(α/2)	O(nlogn)	[25]
α=2π/3	Fixed Orientation	5	O(nlogn)	[26]
2π/3<α<π	Fixed Orientation	2cos(α/2)+2	$O(n^{2})$	[25]
π≤α≤5π/3	Fixed Orientation	2sin(α/2)+1	O(nlogn)	[23]

**Table 2:** Parameters for simulation evaluations.

Parameter	Network Type	
	UADSNs	UASNs
Connection Algorithm	MNCTO	UDG
Deployment Range	1000 × 1000	1000 × 1000
Number of Nodes	100–400	100–400
Clustering Ratio	0.2–1.0	-
Transmission Range	-	[150, 200, 250, 300]
Unit Distance Energy Cost	1	14.93
Routing Aigorithm	Dijkstra	Dijkstra

**Figure A1:**
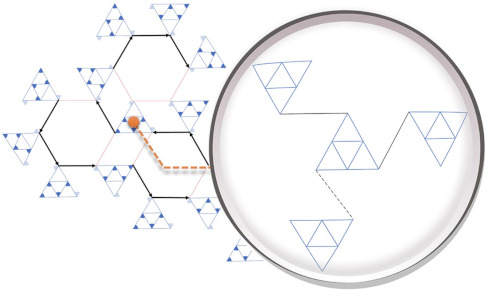
Equilateral triangular widgets connections with others.

## Data Availability

Not applicable.

## Appendix A

**Lemma** **A1.**
*An equilateral triangular widget can connect adjacent edges in a hexagon grid graph.*

**Proof.** From Figure 7a, it can be seen that there are totally 6 variants of equilateral triangular widget, whose orientations are one of the sectors set as S = [[2, 6], [3, 1], [4, 2], [5, 3], [6, 4], [5, 1]] in a counter-clockwise direction. Obviously, their orientations cover the whole Euclidean plane. In addition, the degree of a vertex is intuitive, at most three in a hexagon grid graph, which is also the most sophisticated condition of how a widget can be linked. As illustrated in Figure A1, the construction also includes the situation of a node degree of two. Without loss of generality, the structure depicted above is used to represent connections in a hexagon grid graph, where the black solid lines indicate an equilateral triangular widget that can only connect to at most two other widgets, because it has only two ContactingNodes. The side’s length is *r*, while the dashed line indicates that the vertex is incapable of connecting to other widgets at present but this is not always so in other variants. The conclusions in [18] show that there is no need to make a widget connected to more than two adjacent widgets to construct a Hamiltonian path, but a capacity to orientate to six directions is necessary, which is satisfied by the structure above. Thus, we make the proof. □

**Lemma** **A2.**
*A HPHGG for TOP of nodes set V with side length r, its total inner cost is [(5n−6)(5Klogr+3Ar−2K)−2(Ar+Klog2)]/5, where $n=∥V∥$.*

**Proof.** From Lemma A1, it is known that a widget can only connect to at most two other widgets, and from Figure 6, we notice an EmittingNode can only connect to one of the ContactingNodes directly, so the distance a signal travels is *r*. When another connection (the connection between the EmittingNode and another ContactingNode) is required, a signal need to relay three times to reach, so the total distance is $4*\frac{r}{2}$; they cannot be multiplied directly, as they are not of a linear relationship. Another observation lies in the fact that the Hamiltonian path is a one-way directed path, so a widget should receive from its previous transmission and transmit to its next hop. For the reason explained above, the link distance between two ContactingNode is the sum of their distances to the EmittingNode, so the distance of the inner widget is $r+4*\frac{r}{2}$. Then, the according cost is obtained as follows:

(A1) $$\begin{array}{cc} c_{in} & =cost\left(r\right)+4*cost\left(\frac{r}{2}\right)\\ \; & =5K\log r+3Ar-2K\log2 \end{array}$$

Considering the particularity of head and tail nodes of the path, the total inner cost of HPHGG for TOP is as follows:

(A2) $$\begin{array}{cc} C_{t} & =\left(n-2\right)*c_{in}+4*cost\left(\frac{r}{2}\right)\\ \; & =(n-2)(5K\log r+3Ar-2K\log2)+4K\log r+2(Ar-K\log2)\\ \; & =\left(n-\frac{6}{5}\right)\left(5K\log r+3Ar-2K\right)-\frac{2}{5}\left(Ar+K\log2\right)\\ \; & =\frac{\left(5n-6)(5K\log r+3Ar-2K)-2(Ar+K\log2\right)}{5} \end{array}$$

Thus, the lemma is proved. □
